# Behavioral Paradigms to Probe Individual Mouse Differences in Value-Based Decision Making

**DOI:** 10.3389/fnins.2019.00050

**Published:** 2019-02-07

**Authors:** Opeyemi O. Alabi, Michael P. Fortunato, Marc V. Fuccillo

**Affiliations:** ^1^Department of Neuroscience, University of Pennsylvania, Philadelphia, PA, United States; ^2^Neuroscience Graduate Group, Perelman School of Medicine, University of Pennsylvania, Philadelphia, PA, United States

**Keywords:** operant behavior, mouse, cost-benefit, economic choice, flexibility, value, decision-making

## Abstract

Value-based decision making relies on distributed neural systems that weigh the benefits of actions against the cost required to obtain a given outcome. Perturbations of these systems are thought to underlie abnormalities in action selection seen across many neuropsychiatric disorders. Genetic tools in mice provide a promising opportunity to explore the cellular components of these systems and their molecular foundations. However, few tasks have been designed that robustly characterize how individual mice integrate differential reward benefits and cost in their selection of actions. Here we present a forced-choice, two-alternative task in which each option is associated with a specific reward outcome, and unique operant contingency. We employed global and individual trial measures to assess the choice patterns and behavioral flexibility of mice in response to differing “choice benefits” (modeled as varying reward magnitude ratios) and different modalities of “choice cost” (modeled as either increasing repetitive motor output to obtain reward or increased delay to reward delivery). We demonstrate that (1) mouse choice is highly sensitive to the relative benefit of outcomes; (2) choice costs are heavily discounted in environments with large discrepancies in relative reward; (3) divergent cost modalities are differentially integrated into action selection; (4) individual mouse sensitivity to reward benefit is correlated with sensitivity to reward costs. These paradigms reveal stable individual animal differences in value-based action selection, thereby providing a foundation for interrogating the neural circuit and molecular pathophysiology of goal-directed dysfunction.

## Bullet Points:

- Novel protocol to reveal stable “trait-like” measures of value-based choice in mice.
- Mice integrate relative rewards and costs associated with obtaining those rewards in selecting future actions.
- Reward costs are heavily discounted in the context of large discrepancies in outcome benefit.
- Both increased operant scheduling and temporal delay to reward decrease the relative value of choice alternatives, but do so in divergent manners.
- Sensitivity to reward benefit is correlated with sensitivity to cost at the individual animal level.

## Introduction

In order to make optimal choices in a complex world, individuals must be sensitive to the costs and benefits of particular actions and integrate those components to holistically control motor output (Floresco et al., 2008; Cox et al., 2015; Louie et al., 2015; Kroemer et al., 2016; Mikhael and Bogacz, 2016; Friedman et al., 2017). As neuroeconomic approaches to decision-making have flourished (Kable and Glimcher, 2007; Montague, 2007; Glimcher, 2014; Khaw et al., 2017), there is increasing interest in the cellular and circuit-level neural mechanisms that support value-based action selection (Tai et al., 2012; Wang et al., 2013; Xiong et al., 2015; Parker et al., 2016; Padoa-Schioppa and Conen, 2017). These directions provide a strong foundation to better understand how physiological differences in reward processing contribute to behavioral diversity. Furthermore, they may eventually inform our conception of neuropsychiatric disorders, which often manifest deficits in value-based choice as major features of their behavioral pathology (Der-Avakian and Markou, 2012; Griffiths et al., 2014; Gold et al., 2015; Solomon et al., 2015; Albrecht et al., 2016; Collins et al., 2017; Hélie et al., 2017; Zald and Treadway, 2017).

A number of model systems have been employed to characterize the behavioral aspects of reward processing as well as the neural circuits mediating value representation in the brain (Samejima et al., 2005; Sugrue et al., 2005; Floresco et al., 2008; Lau and Glimcher, 2008; Gan et al., 2010; Sul et al., 2010; Cai et al., 2011; Levy and Glimcher, 2012; Tachibana and Hikosaka, 2012; Wang et al., 2013; Khaw et al., 2017). While the “gold-standard” for these cognitive studies has long been primates (Sugrue et al., 2005; Gold and Shadlen, 2007), rodents offer numerous advantages for pharmacological, cell-type, and circuit-specific molecular approaches (Carandini and Churchland, 2013; Jaramillo and Zador, 2014). Accordingly, behavioral approaches in rats have significantly informed our understanding of the interplay between reward processing and choice. While too numerous to cover here, paradigms such as devaluation, reversal learning, delayed-discounting, operant response scheduling, and probabilistic reward tasks have been employed to examine value encoding, response flexibility, time-dependent value decay, willingness to work, and choice patterns under uncertainty, respectively (Yin et al., 2004, 2005b; Floresco et al., 2008; Castañé et al., 2010; Koffarnus et al., 2011; Mar et al., 2011; Hosking et al., 2015; Yates and Bardo, 2017). Manipulations and recordings done in the context of these behavioral models have begun to reveal the contribution of distinct brain regions to aspects of value processing and goal-directed decision making (Yin et al., 2004, 2005a, 2006; Jones et al., 2012; Gourley et al., 2016; Hart et al., 2018).

The use of mice to characterize economic choice behavior has thus far received less attention. While there have been doubts about the ability of mice to perform the complex cognitive tasks required to assess value-based choice (Carandini and Churchland, 2013; Jaramillo and Zador, 2014), recent work contradicts this idea (Tai et al., 2012; Parker et al., 2016; Guo et al., 2018). As in rat, choice selection under outcome uncertainty has successfully been modeled with alternatives of varying reward probability (Ineichen et al., 2012; Tai et al., 2012; Parker et al., 2016). In addition, the integration of choice benefit and cost has been explored within the context of delayed discounting, whereby larger reward volumes are associated with longer temporal delay to reward delivery (Oberlin and Grahame, 2009; Boomhower and Newland, 2016; Pope et al., 2016). A related attempt at quantifying the discounting of benefit took advantage of the natural tendency for mice to avoid brightly lit spaces as a fixed cost against which rising benefits were compared (Friedman et al., 2017). In our work, we sought to systematically investigate how benefits and two distinct types of action-associated cost are integrated to regulate action selection, with a specific focus on individual mouse differences. Toward this end, we developed a trial-based, forced-choice, serial reversal paradigm that forces mice to make sequential decisions by using previous reward history to continually update subjective choice values. To characterize the subjective value of actions, we measured response bias and performance across a wide dynamic range of reward outcomes and contingencies.

We demonstrate that mice behaviorally manifest internal representations of value by altering the distribution and execution of their choices in response to previously rewarded outcome magnitudes. Furthermore, we show that two unique cost modalities, increased effort to reward and increased delay to reward, generate similar devalued responses but integrate into decision-making via divergent choice mechanisms. Finally, longitudinal, cross-session analysis of individual animal value-processing revealed stable patterns of behavioral performance, consistent with reproducible “trait-like” responses to reward, effort and delay. Together, these findings represent a robust behavioral approach for understanding circuit control of value-based choice in normal and disease-modeled states.

## Materials and Methods

### Animal Subjects

Animal procedures were approved by the University of Pennsylvania Laboratory Animal Care and Use Committee and carried out in accordance with National Institutes of Health standards. Twenty-four adult male C57Bl6/J mice (The Jackson Laboratory, stock# 000664) were used in this study. Animals were housed in cages of at least four, with *ad libitum* access to water. Mice were food-deprived to 85–90% of normal body weight and maintained at this level for the duration of experiments. On days in which no experiments were conducted, mice were weighed and allocated 0.2 g extra chow relative to their recent daily allowance to account for differences in caloric intake between experimental and non-experimental days. Mice were given supplemental food if their weight fell below 85% of their initial weight. Mice were kept on a 7 a.m.–7 p.m. regular light-dark cycle and maintained in constant temperature and humidity conditions.

### Behavioral Apparatus and Task Structure

All experiments were conducted inside a modular chamber with dimensions 8.5 × 7.12 × 5 inches (W × D × H) (Med Associates, Inc., Burlington, VT). Each chamber contained a modified reward magazine through which liquid reward was pumped directly into a custom-made receptacle. On either side of the magazine were retractable levers which had to be fully depressed to register choices. A light in the magazine turned on to indicate the beginning of each trial, after which animals were required to make a sustained (200 ms) magazine head entry to initiate the choice period. The choice period was marked by the extension of levers on either side of the reward magazine, illumination of lights immediately above the protracted levers, and extinction of the magazine light. Mice then had an x-sec temporal window (contingent on current protocol) to register choice via lever press, after which the lever retracted and the trial was considered an omission. Following successful choice selection, the levers were retracted and a variable volume of liquid reward (Boost, 70%, Nestlé) was delivered via the center magazine, which had its light turned on for the duration of the reward period. Reward volumes were determined by variable activation time of single-speed syringe pumps (pre-determined for each pump in prior calibration sessions, Med Associates). Mice were allowed 5 s for reward consumption after which all box lights were inactive for a 1 s inter-trial interval prior to next trial start. All magazine entries (detected by interruption of infrared beam) and lever presses were recorded by MedAssociates software (MedPC-IV). Data were exported to Microsoft Excel via MedAssociates software (MED-PC to Excel).

### Simple Action Outcome Contingency

In the first stage of training, animals were habituated to behavioral boxes for 10 min., followed by a program that delivered 10 μL of reward every minute for 40 min. via the magazine port. Reward delivery was not contingent on mouse choice. Upon reward delivery, the magazine light turned on for 10 s to cue the mouse to reward, followed by a 50 s inter-trial interval. After 3 days in this introductory program, mice learned a lever press-reward contingency. Trials were initiated as described previously. During the choice phase, 1 of 2 levers were protracted, at random, on each trial. Mice had a 10 s temporal window to register their choice via lever press, otherwise the lever retracted and the trial was considered an omission. If animals registered a selection within the given choice time, 10 μL of reward was delivered (P_rew_ = 1.0). Sessions lasted 45 min with no trial number limits. After nine sessions, mice that had completed 2 consecutive days of >150 trials or 1 day >200 trials progressed to the serial reversal task. If mice missed this deadline, they were again assessed after sessions 12 and 15. Mice that failed to meet these criteria by session 15 were excluded from the study (*n* = 3).

### Serial Reversal Task

Animals that met the criteria for acquisition of the action-outcome contingency progressed to a forced-choice two-alternative serial reversal paradigm. Trials began as in the previous protocol, with illumination of the magazine light. Again, mice initiated trials with a 200-ms sustained magazine entry, which led to the choice period. Mice then had a 5 s temporal window to register their choice via lever press, otherwise the lever retracted and the trial was considered an omission. On every trial, both levers were presented. Reward volumes were varied according to experiment and reward probabilities (P_rew_ = 1.0, 0.7, 0.4) were equally applied to both levers. These contingencies were held constant for the duration of a session. Following choice selection, both levers retracted and the 5 s reward phase initiated.

**Figure 1:**
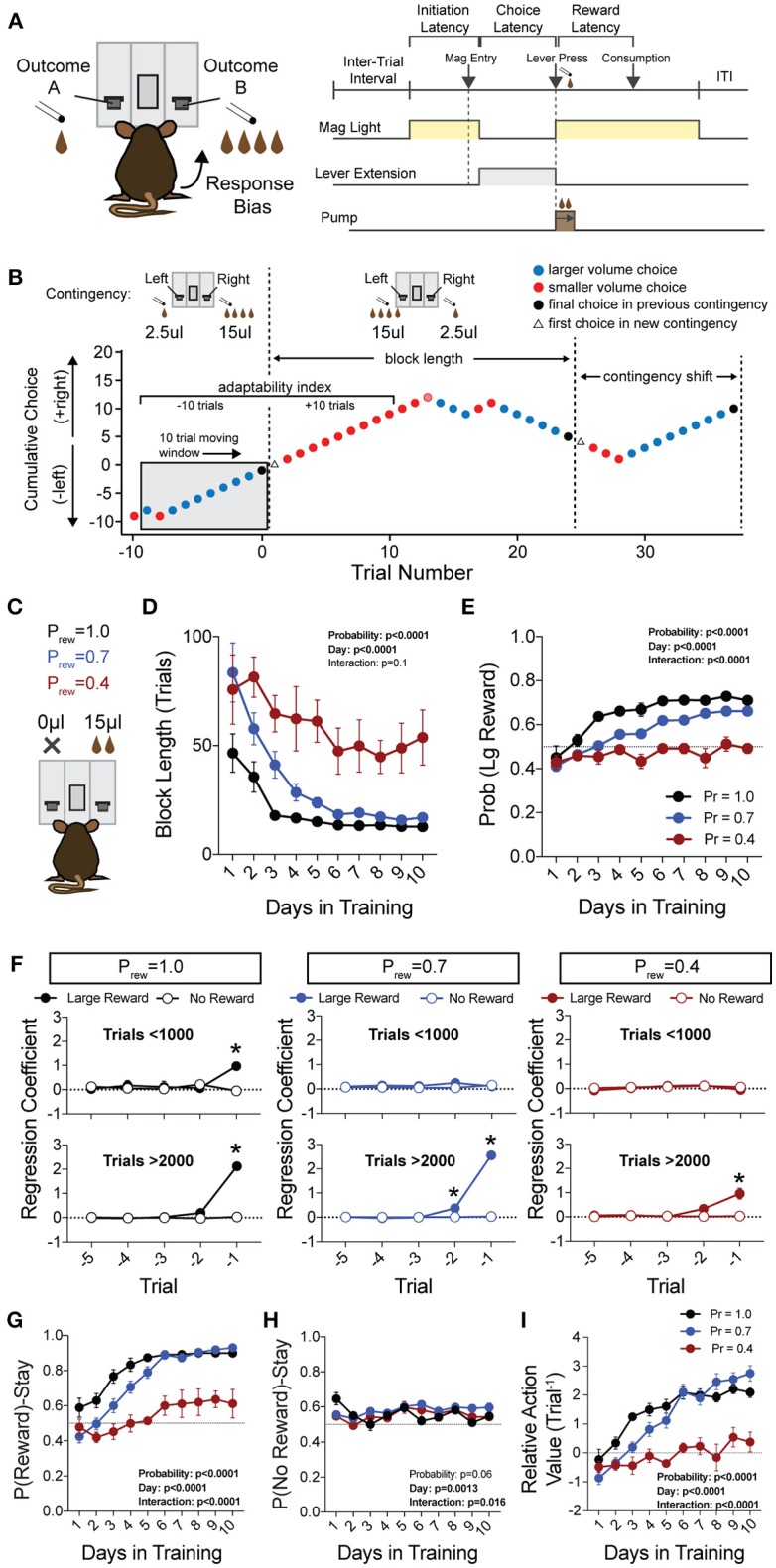
Acquisition of value-based choice paradigm is accompanied by dynamic changes in reinforcement. **(A)** Schematic of trial structure showing that mice initiate trials via sustained magazine entry, respond via lever press during specified temporal window, and collect rewards from center magazine. **(B)** Block structure—trials with the same contingency occur consecutively until mice select the alternative with the large reward eight times in a proximal window of 10 trials. Dotted lines signify block switch and gray box denotes 10-trial moving window for triggering contingency switch. **(C)** Mice that were trained in a simple lever press-reward contingency were initiated into the reversal paradigm at one of three probabilities of reinforcement. On any given trial, one alternative resulted in reward and the other resulted in no reward (P_rew_ = 1, *n* = 5; P_rew_ = 0.7, *n* = 11; P_rew_ = 0.4, *n* = 5). **(D)** Block length, the average number of trials until a contingency switch, decreased over the duration of the training period in a reward probability dependent fashion. **(E)** The overall probability that mice select the large reward increases over the duration of training in a reward probability dependent fashion. **(F)** Logistic regression modeled the effects of past reinforcers on subsequent choice in early (<1,000 trials, top) and late (>2,000 trials, bottom) periods of acquisition. We performed multiple *t*-tests comparing “Large Reward” and “No Reward” coefficients to assess which reward outcome types were reinforcing (significance indicated by asterisk, corrected for multiple comparisons using Holm-Sidak). In early acquisition, only the T^−1^ trial at P_rew_ = 1 was positively reinforcing relative to no reward outcome. In later acquisition, the T^−1^ trial was significantly reinforcing for all probabilities tested. **(G)** The probability that animals stayed on a choice alternative after receiving a large reward (Pr(Reward)-Stay) increased over the course of training in a reward probability dependent manner. **(H)** There was a significant effect of acquisition day on the probability that animals stayed on a choice alternative after receiving no reward (Pr(No Reward)-Stay), however, pairwise comparisons revealed few differences in these values and we noted no consistent differences over the course of multiple days. **(I)** The relative action value, defined as the reinforcing property of large reward vs. no reward outcomes, increased as animals gain experience in this paradigm in a reward probability dependent fashion. All data analyzed by Repeated Measures (Day) Two-Way ANOVA.

To prevent outcome-insensitive behavior, we employed a “moving window” to trigger changes in lever-reward association (Figure 1B). When 8 of the last 10 actions were allocated to the large reward volume side, an un-cued contingency shift flipped the lateralization of the high and low benefit alternatives. The probability of reinforcement as well as the relative reward contrast between choices were kept consistent over individual sessions. Sessions were limited to 1 h, or 360 trials, whichever occurred first. Each relative reward contingency was performed on the same animal in a semi-random order (contingencies were never repeated on adjacent days).

### Application of Response Costs

We decided to model costs as operant contingencies that either increased the number of required operant responses or the temporal delay prior to reward delivery. Costs were exclusively associated with the large reward benefit alternative in all contingencies. In tasks in which repetitive motor output was required, selection of an alternative led to retraction of the unselected lever and extinction of the corresponding lever light. The selected alternative remained protracted until the animal completed the required motor repetitions. In tasks in which a temporal disparity was introduced between choice and outcome, selection of an alternative initiated the requisite time disparity, with no light indicating the presence of reward. Upon completion of the time delay, the reward period was triggered with the illumination of magazine light and delivery of the appropriate volume of reward. Each cost-benefit contingency was performed on the same animal in a semi-random order (contingencies were never repeated on adjacent days).

### Analysis of Behavioral Performance

Data were analyzed using custom-written scripts developed in *R Studio* (3.3.1) (R Core Team, 2017), making use of base functions supplemented by Rmisc (Hope, 2013), plyr (Wickham, 2011), and reshape2 (Wickham, 2007) packages. All analysis code is freely available upon request.

The Average Block Length over the course of a session was calculated as:

$$\begin{array}{l} Average\;Block\;Length=\left(\overset{n}{\underset{i=1}{\sum }}BL_{i}\right)/n \end{array}$$

where *BL*_*i*_ refers to the number of trials till a contingency switch in the *i*th block of an individual session and n is the number of blocks completed in a session.

Overall session performance was calculated as:

$$\begin{array}{l} Prob\left(Large\;Reward\right)=\frac{C_{Large}}{C_{Total}} \end{array}$$

where *C*_*Large*_ and *C*_*Total*_ refer to the total number of choices to the large reward alternative and the total number of choices made in an individual session, respectively.

The Relative Action Value of a larger volume outcome, A, vs. a smaller volume outcome, B, was calculated as:

$$\begin{array}{l} Relative\;Action\;Value=\ln\left(\frac{\left(\frac{Pr\left(A\right)}{1-Pr\left(A\right)}\right)}{\left(\frac{Pr\left(B\right)}{1-Pr\left(B\right)}\right)}\right) \end{array}$$

where Pr(A) and Pr(B) refer to the probability that mice stay on the choice alternative producing the larger volume outcome (A) and the smaller volume outcome (B), respectively, on the T^−1^ trial.

Adaptability Index was calculated as:

$$\begin{array}{l} AdaptabilityIndex=\left(\overset{n}{\underset{i=1}{\sum }}\frac{\left(L_{i}^{post}-S_{i}^{post}\right)+\;\left(L_{i}^{pre}-S_{i}^{pre}\right)}{10}\right)/n \end{array}$$

where $L_{i}^{pre}$ and $L_{i}^{post}$ refer to the number of large alternative selections in the 10 trials before and after the *i*th contingency switch in an individual session and $S_{i}^{pre}$ and $S_{i}^{post}$ refer to the number of small alternative selections in the same time window. n is the number of blocks completed in a session.

Relative Initiation Latency was calculated as:

$$\begin{array}{l} RelativeLatencytoInitiate=\;\frac{LatInit_{Large}-LatInit_{Small}}{LatInit_{Small}} \end{array}$$

where LatInit_Large_ and LatInit_Small_ refer to the average latency to initiate trials following large reward and small reward outcomes, respectively, in an individual session.

The extent to which the application of reward costs affected the choice distribution of individual animals was calculated as:

$$\begin{array}{l} {Cost\;Discounting}_{Individual}=\;RAV_{Cost}-\;RAV_{NoCost} \end{array}$$

where *RAV*_*Cost*_ and *RAV*_*NoCost*_ refer to the relative action values for animals in reward contingencies with and without cost considerations, respectively. The magnitude of values represent the degree to which costs decrease (negative values) or increase (positive values) the relative value of choice alternatives to which costs have been applied. The relative sensitivity of animals to particular cost modalities was calculated as the z-score of this value.

A logistic regression model was used to model current choice as a function of past actions (*n* = 5 trials) and their resulting outcomes:

$$\begin{array}{lll} \log\left(\frac{R\left(i\right)}{1-R\left(i\right)}\right)=\beta_{0} & + & \overset{n}{\underset{p=1}{\sum }}\beta_{p}^{LR}LR\left(i-p\right)+\;\overset{n}{\underset{p=1}{\sum }}\beta_{p}^{SR}SR\left(i-p\right)\\ + & \;\overset{n}{\underset{p=1}{\sum }}\beta_{p}^{NR}NR\left(i-p\right)+error \end{array}$$

where *R*(*i*) is the probability of choosing the right-sided alternative on the *i*th, current, trial. LR(*i-p*), SR(*i-p*), and NR(*i-p*) refer to the outcomes of the *p*th previous trial. LR(*i-p*) is defined such that LR(*i-p*) = +1 if an animal received a large reward on the *p*th previous trial resulting from a right press, a −1 if an animal received a large reward on the *p*th previous trial resulting from a left press and 0 if the animal did not receive a large reward. SR(*i-p*) and NR(*i-p*) are defined similarly for trials that resulted in small reward and no reward outcomes, respectively. Together these variables account for lateralization of past choices and the resultant outcomes. This method assumes equivalent reinforcement from outcomes regardless of the lateralization of choice. Regression coefficients were fit to individual mouse data using the *glm* function in R with the binomial error distribution family. Coefficient values for individual mice were averaged to generate the plots and perform the analysis observed in Figure 1F and **Figure 4**.

### Reinforcement Learning Model

We fit an adapted Q-Learning Reinforcement Model with four parameters to our behavioral data (Sutton and Barto, 1998; Daw, 2011). The input to this model was the sequence of choices of each mouse and resulting outcomes. Similar to our logistic regression model, the reinforcement model assumes that choice behavior is heavily influenced by the subjective value of each alternative on a given trial. Action values for the two alternatives were initiated at 0 and values were updated via the following rule:

$$\begin{array}{l} Q_{t+1}=Q_{t}+\;\alpha \left(R_{t}-Q_{t}\right) \end{array}$$

where *Q*_*t*_ is the value of the action taken on trial *t* and *R*_*t*_ is the reward volume resulting from that action. *R*_*t*_ − *Q*_*t*_ thus defines the reward prediction error on a given trial and this value, scaled by the learning rate (α), is used to update the value of subsequent actions. In this context, α determines the extent to which new information about the state-action pairing alters subsequent behavior. In keeping with standard Q-learning models, values for the unchosen alternative were not updated. In order to model the choice behavior of mice based on these action values, we implemented a softmax decision function to convert values into action probabilities. The operator computed the probability of choosing an alternative A on trial t as:

$$\begin{array}{l} P_{A}\left(t\right)=\;\frac{1}{1+\;e^{-z}}\;,where\\ z=\;\beta \left(Q_{A}\left(t\right)-Q_{B}\left(t\right)\right)+\;\kappa C_{t-1}+c \end{array}$$

The inverse temperature parameter, β, is the regression weight linking the values of each option to the choice output. High values for β indicate mice more readily exploit differences in action values between the alternatives, while lower values suggest that mice exhibit more exploratory behavior. To account for the global preference or aversion of mice to choices they have recently made, we included a term, κ*C*_*t*−1_, where *C*_*t*−1_ is an indicator variable that denotes whether the animal selected alternative A on the previous trial (*C*_*t*−1_ = 1 if animal selected choice A and = −1 otherwise). κ represents the extent to which the animal's previous choice influences its subsequent choice irrespective of outcome. The constant value, c, captures any existing preference for a particular choice alternative. In order to fit this model to our choice data we performed a maximum likelihood fit using function minimization routines of the negative log likelihood of models comprised of different combinations of our three parameters (α, β, κ, c) in MATLAB (Vo et al., 2014).

### Statistical Methodology

All data were initially tested with appropriate repeated measure ANOVA (Prism 7.0). Main effect and interaction terms are described both within figures and accompanying figure legends. Results of relevant *post-hoc* testing (Tukey's multiple comparisons) are included in Table 1.

**Table 1:** Statistical results of pairwise *post-hoc* comparisons for: Figure 1H—means of Pr(NO REWARD)-Stay for specified days; Figure 3D—means of Pr(LARGE REWARD)-Stay for different reward contrast and probability; Figure 3G—means of Latency to Initiate for different reward contrast and probability; Figure 8E—means of Pr(SMALL REWARD)-Stay for different temporal delays.

	**Mean 1**	**Mean 2**	**Mean difference**	**Adjusted *P*-Value**	**Significance**
**FIGURE 1H****: Pr(NO REWARD)-STAY**					
1 vs. 2	0.575	0.528	0.047	0.135	ns
1 vs. 3	0.575	0.549	0.026	0.864	ns
1 vs. 4	0.575	0.555	0.021	0.967	ns
1 vs. 5	0.575	0.599	−0.024	0.923	ns
1 vs. 6	0.575	0.584	−0.009	1.000	ns
1 vs. 7	0.575	0.564	0.011	1.000	ns
1 vs. 8	0.575	0.591	−0.016	0.995	ns
1 vs. 9	0.575	0.561	0.014	0.998	ns
1 vs. 10	0.575	0.573	0.003	1.000	ns
2 vs. 3	0.528	0.549	−0.021	0.959	ns
2 vs. 4	0.528	0.555	−0.027	0.846	ns
2 vs. 5	0.528	0.599	−0.071	0.002	^**^
2 vs. 6	0.528	0.584	−0.056	0.032	^*^
2 vs. 7	0.528	0.564	−0.036	0.481	ns
2 vs. 8	0.528	0.591	−0.063	0.008	^**^
2 vs. 9	0.528	0.561	−0.033	0.615	ns
2 vs. 10	0.528	0.573	−0.044	0.198	ns
3 vs. 4	0.549	0.555	−0.006	1.000	ns
3 vs. 5	0.549	0.599	−0.050	0.095	ns
3 vs. 6	0.549	0.584	−0.035	0.533	ns
3 vs. 7	0.549	0.564	−0.015	0.996	ns
3 vs. 8	0.549	0.591	−0.042	0.275	ns
3 vs. 9	0.549	0.561	−0.012	0.999	ns
3 vs. 10	0.549	0.573	−0.023	0.927	ns
4 vs. 5	0.555	0.599	−0.044	0.209	ns
4 vs. 6	0.555	0.584	−0.029	0.758	ns
4 vs. 7	0.555	0.564	−0.009	1.000	ns
4 vs. 8	0.555	0.591	−0.036	0.483	ns
4 vs. 9	0.555	0.561	−0.006	1.000	ns
4 vs. 10	0.555	0.573	−0.018	0.988	ns
5 vs. 6	0.599	0.584	0.015	0.997	ns
5 vs. 7	0.599	0.564	0.035	0.551	ns
5 vs. 8	0.599	0.591	0.008	1.000	ns
5 vs. 9	0.599	0.561	0.038	0.420	ns
5 vs. 10	0.599	0.573	0.026	0.857	ns
6 vs. 7	0.584	0.564	0.020	0.973	ns
6 vs. 8	0.584	0.591	−0.007	1.000	ns
6 vs. 9	0.584	0.561	0.023	0.930	ns
6 vs. 10	0.584	0.573	0.012	1.000	ns
7 vs. 8	0.564	0.591	−0.027	0.847	ns
7 vs. 9	0.564	0.561	0.003	1.000	ns
7 vs. 10	0.564	0.573	−0.008	1.000	ns
8 vs. 9	0.591	0.561	0.030	0.740	ns
8 vs. 10	0.591	0.573	0.018	0.983	ns
9 vs. 10	0.561	0.573	−0.011	1.000	ns
**FIGURE 2D****: Pr(LARGE REWARD)-STAY**					
P_rew_ = 0.7				
15/10 μL vs. 15/5 μL	0.928	0.959	−0.031	0.150	ns
15/10 μL vs. 15/0 μL	0.928	0.958	−0.030	0.174	ns
15/5 μL vs. 15/0 μL	0.959	0.958	0.001	0.996	ns
P_rew_ = 0.4				
15/10 μL vs. 15/5 μL	0.877	0.880	−0.003	0.984	ns
15/10 μL vs. 15/0 μL	0.877	0.842	0.035	0.113	ns
15/5 μL vs. 15/0 μL	0.880	0.842	0.038	0.080	ns
**FIGURE 2G****: LATENCY To INITIATE**					
P_rew_ = 0.7				
15/10 μL vs. 15/5 μL	1.194	1.113	0.081	0.932	ns
15/10 μL vs. 15/0 μL	1.194	1.318	−0.124	0.850	ns
15/5 μL vs. 15/0 μL	1.113	1.318	−0.205	0.642	ns
P_rew_ = 0.4				
15/10 μL vs. 15/5 μL	1.507	2.094	−0.587	0.047	^*^
15/10 μL vs. 15/0 μL	1.507	2.696	−1.189	<0.001	^****^
15/5 μL vs. 15/0 μL	2.094	2.696	−0.602	0.041	^*^
**FIGURE 5E****: Pr(SMALL REWARD)-STAY**					
0 s vs. 1.5 s	0.804	0.813	−0.009	0.927	ns
0 s vs. 3 s	0.804	0.807	−0.003	0.996	ns
0 s vs. 4.5 s	0.804	0.867	−0.064	0.000	^***^
1.5 s vs. 3 s	0.813	0.807	0.006	0.979	ns
1.5 s vs. 4.5 s	0.813	0.867	−0.055	0.002	^**^
3 s vs. 4.5 s	0.807	0.867	−0.061	0.000	^***^

* P ≤ 0.05;

** P ≤ 0.01;

*** P ≤ 0.001;

**** *P ≤ 0.0001*.

## Results

### A Dynamic Choice Paradigm to Probe Value-Based Behavioral Selection

**Figure 2:**
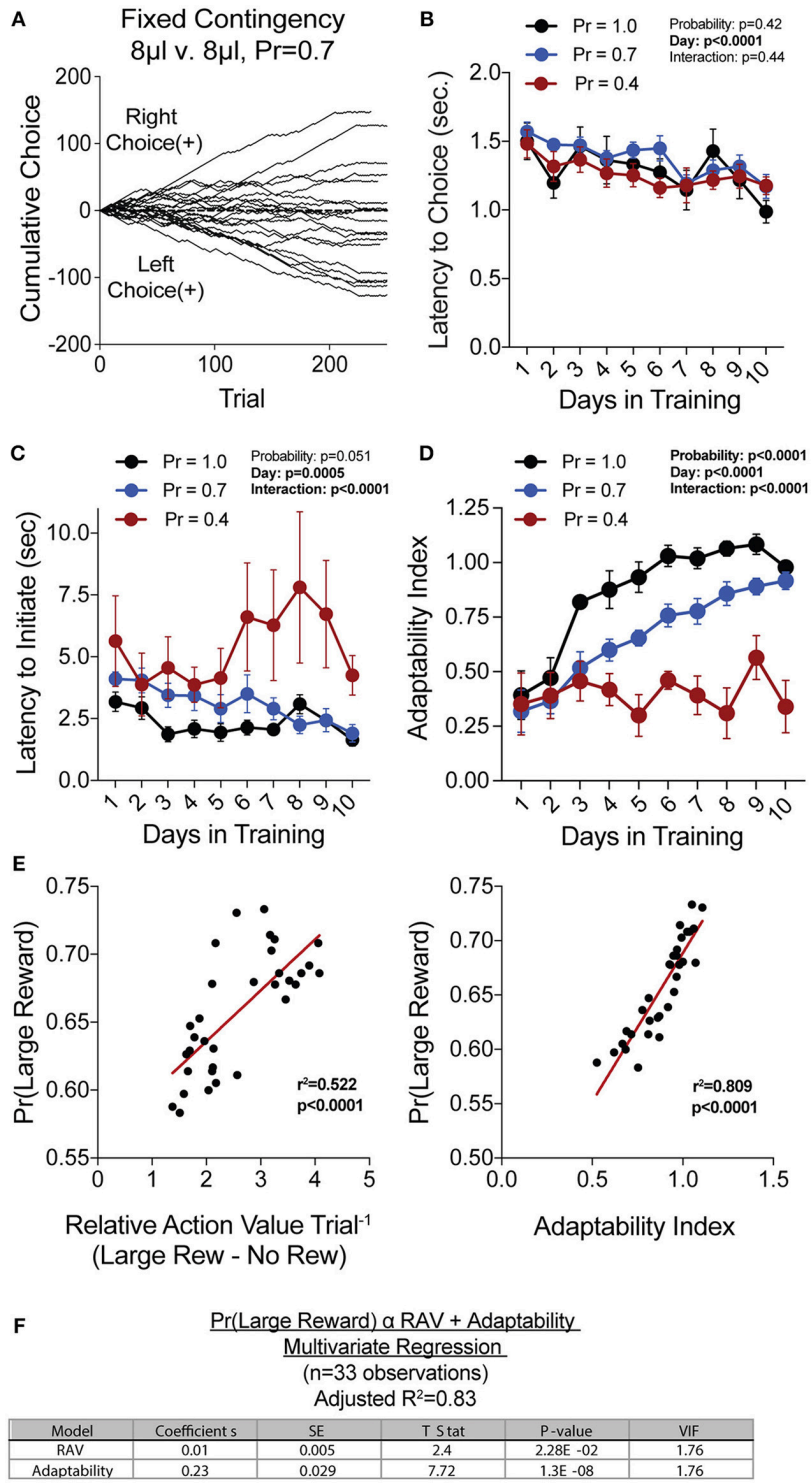
Acquisition of value-based choice paradigm is accompanied by dynamic changes in motor-efficiency and behavioral flexibility: **(A)** Choice patterns in the absence of benefit differences show that some mice continually sample the two available options while others develop significant choice bias. **(B,C)** As animal acquire the reversal task, they display increased motor efficiency in the execution of the task, including the speed with which choices are made [latency to choice **(B)**] and the speed with which trials are initiated [latency to initiate **(C)**]. **(D)** Adaptability, a measure of choice flexibility, shows a probability-dependent increase with training. **(E)** Both the relative action value (left) and the adaptability index (right) have significant linear relationships with overall task performance in an individual session (data from Days 8,9,10 of *n* = 11 mice at P_rew_ = 0.7) **(F)** Bivariate linear regression analysis of session performance against RAV and the Adaptability Index indicates that both of these variables are significant, with minor multicollinearity (VIF = 1.76). Together, they account for 83% of variability in session performance of mice.

To generate a reliable global estimation of how individual animals weighed benefits and costs, we employed a block structure design that maximized trial number while preventing outcome-insensitive behavior by dynamically altering reward contingencies in response to proximal choice patterns (Methods, Figures 1A,B, see outcome-insensitive effects of fixed contingency in Figure 2A). After acquiring a simple lever press-for-reward contingency, mice progressed to the dynamic reversal task. For initial training, one lever was associated with 15 μL of reward while the other was unrewarded (Figure 1C). Furthermore, feedback density was manipulated by applying reward probabilities to both choices (P_rew_ = 1.0, 0.7, and 0.4 for different cohorts of mice throughout training). The number of trials performed between contingency switches (Block Length) as well as the overall probability that mice chose the rewarded outcome [Pr(Large Reward)] served as global measures of choice efficiency in this task (Figure 1B). We observed that these two measures tracked in opposite directions over 10 days of training, with the average block length steadily decreasing (Figure 1D), and the overall rate of large reward selection steadily increasing (Figure 1E), both in a reward probability-dependent manner. Consistent with increased global task efficiency, we observed a reduction in choice and initiation latencies (Figures 2B,C).

To understand the changes in action selection underlying this increased behavioral efficiency, we explored how reward differentially shaped behavior in early and late periods of training. When first presented with a choice between levers following rewarded outcomes, mice exhibited random choice preference. Upon further training, these distributions became biased toward choices that produced proximal rewarded outcomes (Figures 1E,G). To further probe the factors influencing mouse decision-making in this task, we employed a logistic regression model (Lau and Glimcher, 2005; Tai et al., 2012; Parker et al., 2016) to quantify how an animal's previous choices and resulting outcomes impacted their subsequent choices during early and late learning. In contrast to models generated from trials in early learning (Figure 1F, top), trials from later periods of training revealed a marked influence of the immediately preceding trial (T^−1^) on future choice (Figure 1F, bottom). The degree to which prior trials contributed to current choice varied according to reward probability, with sparse conditions (P_rew_ = 0.4) driving less robust control of current behavior. Given that the outcome of the T^−1^ trial largely determined future choice for higher rates of reinforcement (P_rew_ = 0.7 and 1; the reward environments for our following experiments), we focused primarily on this trial for analyses of behavior.

We hypothesized that overall efficiency in this task would be driven by increasing ability to (a) choose the higher value option and (b) flexibly alter behavioral responding at contingency changes. To better characterize how different outcome values alter mouse choice patterns, we systematically compared the probability that animals returned to a choice after receiving a reward (reward-stay, Figure 1G) with the probability of returning after receiving no reward (no reward-stay = [1-(no reward-shift)], Figure 1H). This measure, which we call the “relative action value,” increased with training and plateaued at ~2.5, representing ~12-fold higher odds of staying on an alternative that produced a 15 μL reward as compared to a no reward alternative (Figure 1I). Relative action value serves as an internally controlled metric describing the comparative reinforcing properties of distinct operant outcomes. To capture how flexible responding contributed to global task efficiency, we compared the choice patterns in the 10 trials before and after un-cued contingency switches (Figure 1B). This adaptability metric progressively increased across 10 days of training, suggesting the mice more dynamically modulated their behavior as they learned the overall task structure (Figure 2D). Furthermore, we noted a consistent trend toward greater flexibility with higher reinforcement probabilities, consistent with the idea that negative feedback signals are particularly relevant for switching behaviors. To test whether these two metrics—relative action value and adaptability—explain global task performance of individual mice, we performed a bivariate linear regression of these metrics against Pr(Large Reward). We chose the last 3 days of training at P_rew_ = 0.7 (*n* = 33 sessions; no mean difference in population performance over these days) to perform this analysis. We found that together, the relative action value and adaptability index explained 82% of the variability in individual session performance (adjusted *R*^2^, Figures 2E,F).

### Modulation of Choice by Relative Benefit

**Figure 3:**
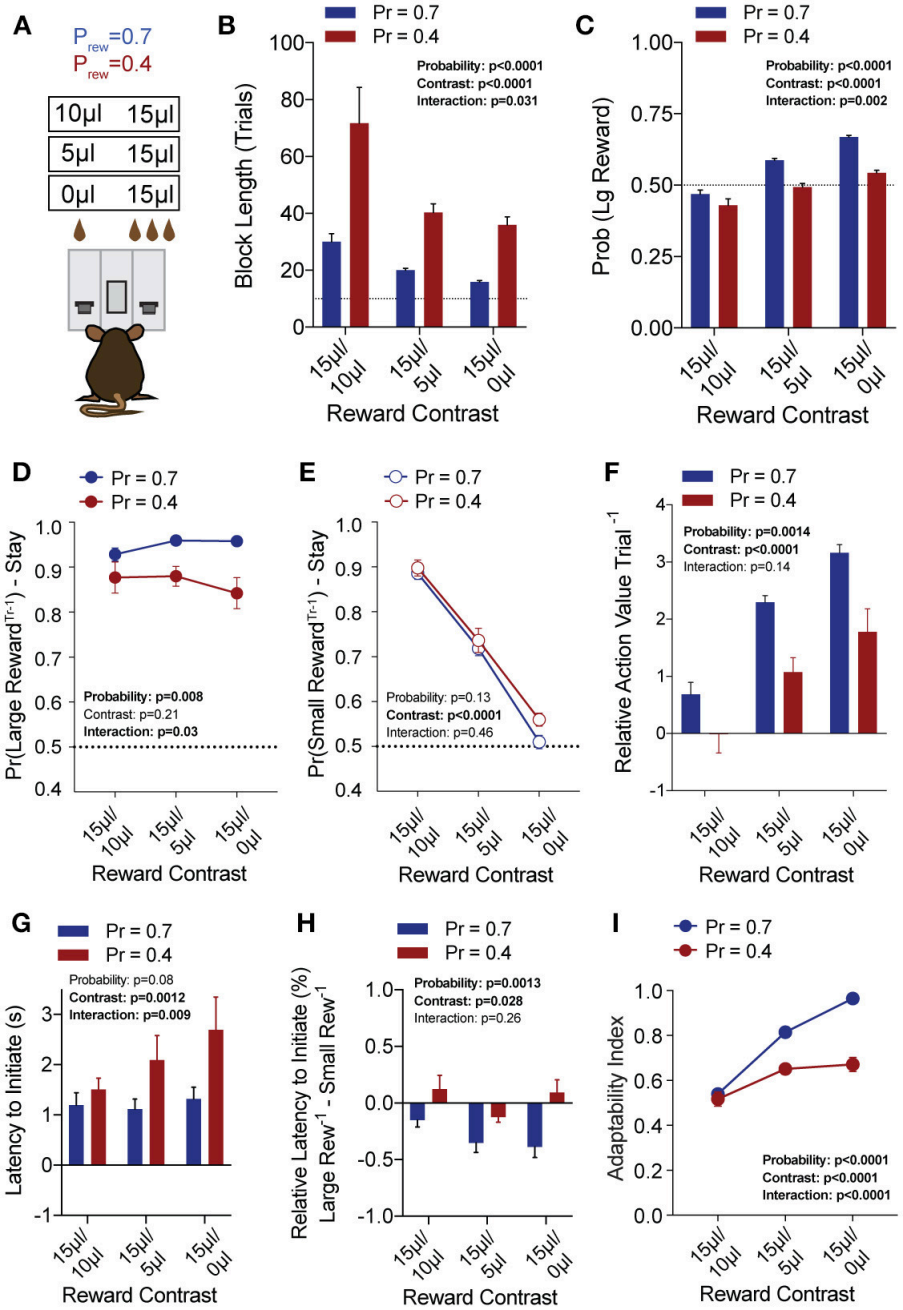
Choice is strongly shaped by differentially rewarded outcomes. **(A)** Animals (*n* = 21) were tested at three reward magnitude contrasts and either high or low reinforcement probability regimes. **(B,C)** The average block length and the probability that animals select the large reward alternative over the course of a session are both sensitive to the relative contrast in reward as well as the probability of reinforcement. **(D)** While there was a significant interaction between reward contrast and probability for Large-Reward-Stay behavior, Tukey's multiple comparisons test revealed no pairwise differences for relative rewards within individual probabilities of reinforcement. **(E)** There was a significant main effect of reward contrast on Small-Reward-Stay behavior. **(F)** The relative action value exhibited significant main effects for both relative reward contrast and probability of reinforcement. **(G)** The latency to initiate trials, averaged by session, showed a significant effect for reward contrast and an interaction between reward contrast and probability. Pairwise differences obtained by Tukey's multiple comparison's test indicate no significant differences in the initiation times for mice at the higher probability of reinforcement. **(H)** The relative initiation latency demonstrates that at P_rew_ = 0.7, mice more rapidly initiate trials following large than small rewards. This disparity is sensitive to relative reward magnitude contrast. **(I)** Behavioral flexibility after contingency switches is sensitive to relative reward magnitude contrasts as well as the probability of reinforcement. All data analyzed by Repeated Measures (Reward Ratio) Two-Way ANOVA.

**Figure 4:**
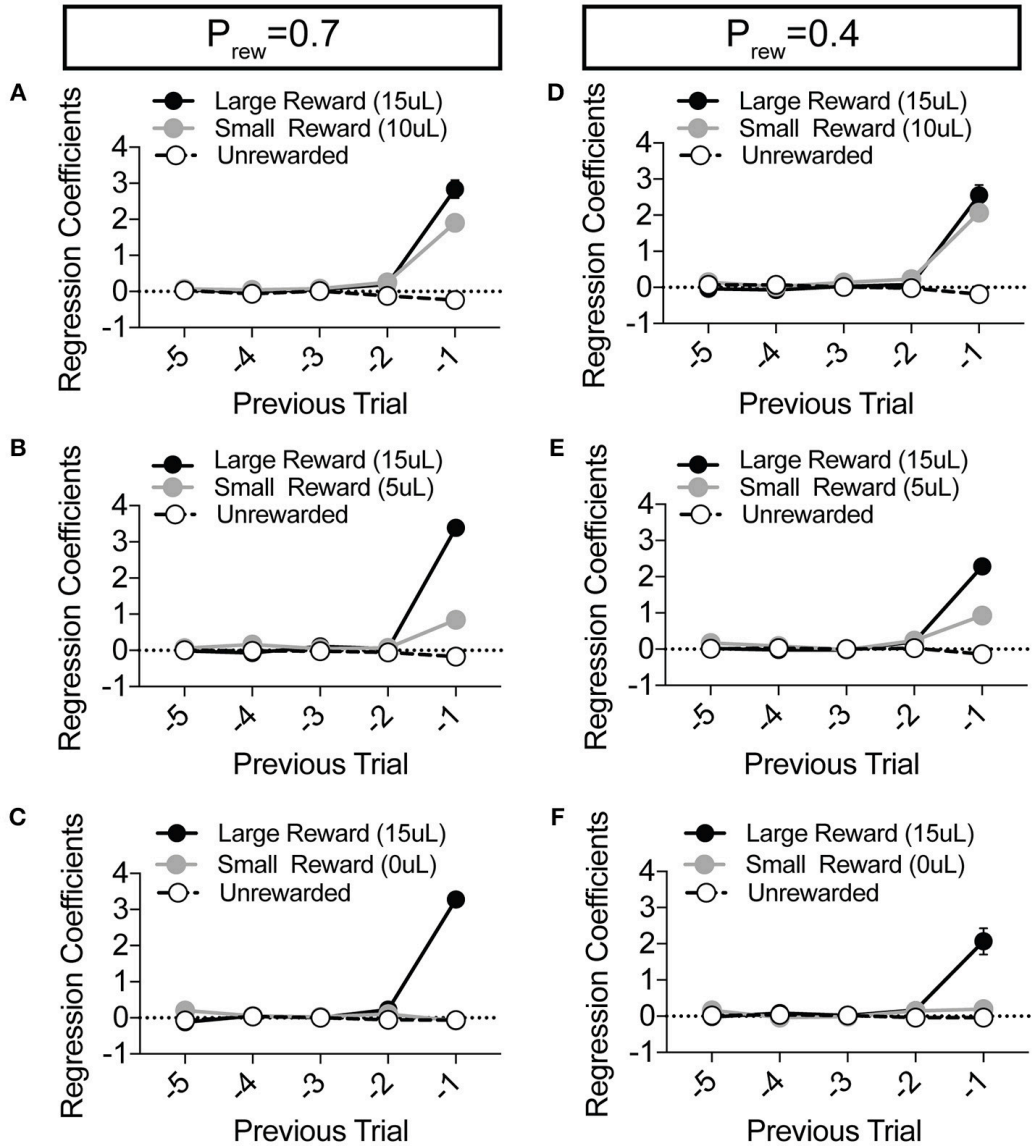
Choice is largely influenced by the T^−1^ trial in a relative reward environment. **(A–F)** The average coefficients for the multivariate logistic regression model that describes previous choice and reward history. (P_rew_ = 0.7, *n* = 11; P_rew_ = 0.4; *n* = 10) For each of the relative reward regimes tested, note that the T^−1^ trial is significant for large reward outcomes. For relative reward regimes with 10 μL **(A,D)** and 5 μL **(B,E)** small reward outcomes we note a significant T^−1^ coefficient. We detect no significant T^−2^ (or further) coefficients indicating that mouse choice is largely dictated by the outcome of the T^−1^ trial.

To explore the sensitivity of mice to differentially beneficial outcomes, we associated each lever with a specific reward volume, keeping the large reward at 15 μl and randomly altering the small reward between 10, 5, and 0 μl in separate sessions (Figure 3A). Data were compiled from three separate sessions per mouse for each contingency. Given that block completion depends on the formation of biased choice patterns, we were unsurprised to find that larger reward contrasts had shorter block lengths and higher overall rates of large reward selection than smaller relative reward contrasts, at both high and low reward probabilities (Figures 3B,C). We modified our logistic regression model to include a term for small rewarded outcomes and the coefficients generated from the behavioral data in each contingency again demonstrated the weight of the immediately preceding trial (T^−1^) (Figure 4), so we focused our analysis on T^−1^ win-stay probabilities for each operant outcome (Figures 3D,E). The relative action values for large vs. small reward outcome demonstrated a stepwise decrease with smaller relative reward contrasts (Figure 3F). While we noted a small but significant effect for reward contrast on the reinforcing properties of the 15 μL reward (Figure 3D, Large Reward-Stay), the alteration in the relative value of large and small rewarded outcomes was mainly driven by the increased reinforcing property of the small reward (Figure 3E). We also noted that for each relative reward environment, decreasing the overall rate of reinforcement decreased the relative value of large vs. small rewarded outcomes, indicating a relationship not just between reward volume and reinforcement, but reward frequency and reinforcement (Figure 3F). Taken together, these data show that within this feedback-directed task, animal choice can be explained as the result of competing reinforcement probabilities that relate most strongly to total reward volume.

In addition to the modulation of lever return by outcome benefit, we also noted a consistent alteration in initiation latencies depending on the previous trial outcome (Figure 3H). While the average initiation latencies of most trials were stable at ~1 s for P_rew_ = 0.7 (Figure 3G), when we sorted trials by their prior outcome we found that mice more rapidly initiated trials following large reward outcomes (Figure 3H, blue bars). This local modulation of action performance, which may provide a proxy for attentional or motivational states (Wang et al., 2013) was seen only for the initiation epoch latency (not shown) and was not robustly observed in sparser reward conditions (Figure 3H, P_rew_ = 0.4). As demonstrated previously, the rate at which animals selected the large reward outcome in this task was influenced not just by the ability of the animals to discriminate between relative benefits of the lever alternatives, but also by their flexibility at contingency switches. Here we note that adaptability is modulated by differing value contingencies (Figure 3I), with a significant interaction between reward magnitude and rate of reinforcement.

**Figure 5:**
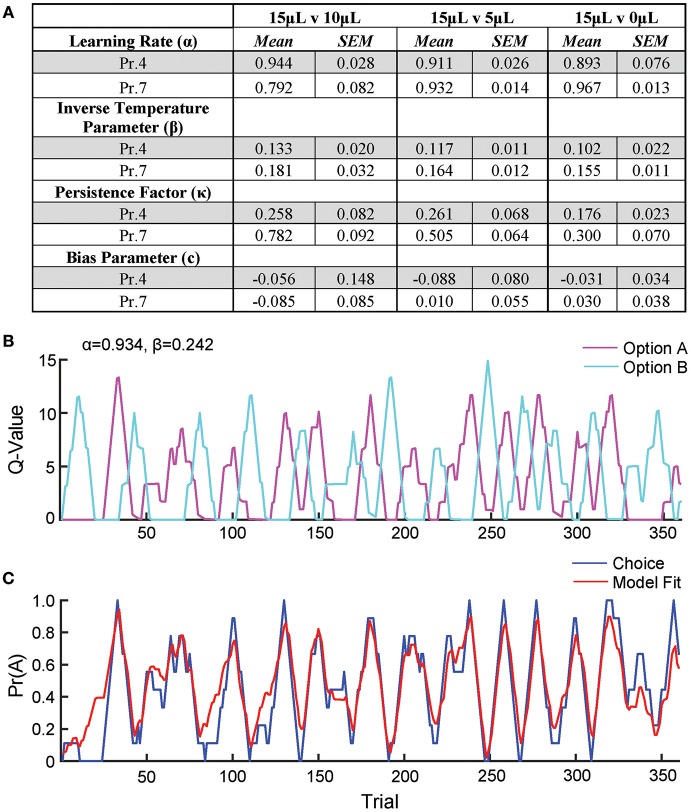
Q-learning reinforcement learning model predicts choice behavior. In order to extract information on how mice update action values using reward prediction errors (α) and how sensitive mouse choice is to differences in action values (β), a reinforcement learning model was fit to the choice data of mice performing the relative reward paradigm. **(A)** Table summarizing model parameters in both reward probability environments. **(B)** The calculated *Q*-values of the two levers for an individual mouse in a single session [15 vs. 0 μL at P_rew_ = 0.7]. As the animal performs reversals, we note an oscillation in the action value for both options. **(C)** The probability that the mouse selected alternative A on a given trial vs. predicted probabilities generated by the full model. This model is a significant predictor of choice behavior (*R*^2^ = 0.43). *Q*-values and choice probabilities are calculated as a moving average of nine trials.

To further describe how mice selected actions based on relative benefit, we fit a reinforcement Q-learning model to our behavioral data (see section Materials and Methods and Figure 5; Sutton and Barto, 1998; Daw, 2011). Our model comprised four principle components of mouse choice behavior—learning rate, inverse temperature parameter, choice persistence factor and bias parameter. The model was fit to choice and reward data by estimating the action value of alternatives on any given trial via a standard iterative Q-learning algorithm, *Q*_*t*+1_ = *Q*_*t*_ + α(*R*_*t*_ − *Q*_*t*_). Here, the learning rate (α) provides a measure of how strongly new reward information modifies values for a specific action. To characterize the extent to which action values influenced choice behavior, we utilized a softmax decision function to solve for the inverse temperature parameter (β). We added additional terms to capture the propensity of mice to repeat actions irrespective of previous outcome, κ, and a constant measure of bias, c, for one alternative over another. We fit this model to our data and observed that it predicted actual mouse choice behavior with high accuracy (Figures 5B,C).

We observe high α values (>0.79) in most reward environments tested, suggesting a high gain for proximal reward history—a finding supported by our regression model of choice behavior (Figures 5A, 1F). Furthermore, we noted a significant effect of reward probability on the value of κ, with higher reinforcement rates encouraging a higher probability of remaining on chosen actions, particularly with small reward contrasts. Interestingly, neither α nor β varied substantially across relative reward contrast or probability of reward (Figure 5A). This suggests that differences in behavioral efficiency and adaptability observed as relative rewards fluctuate (Figures 3B,C) do not exclusively result from gross changes in reward-seeking strategy between sessions.

### Stable Characteristics of Reward Processing

**Figure 6:**
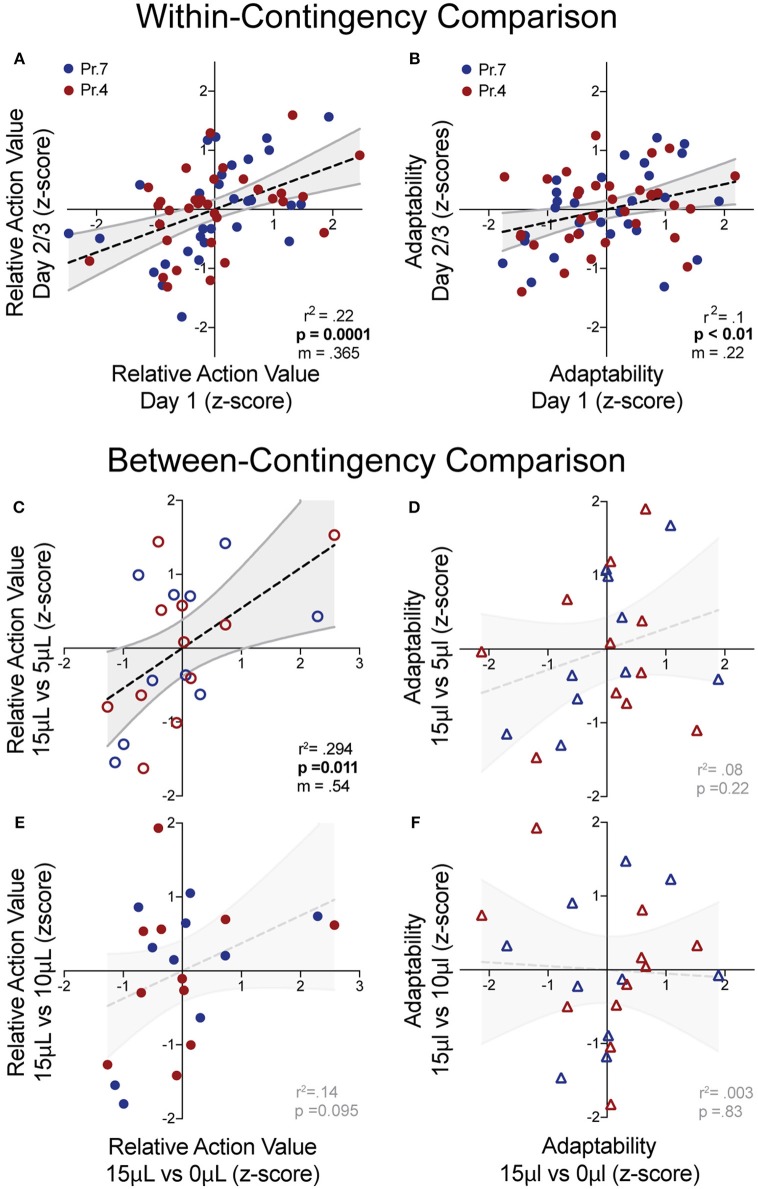
“Trait-like” stability of reward sensitivity and flexibility measures. **(A,B)** Cross-session correlation of relative action value and adaptability index revealed a significant positive linear relationship between the values of mice relative to the population on Day 1 and the values of mice relative to the population on Days 2/3 (averaged) for both reward sensitivity and behavioral flexibility. **(C,D)** Cross-contingency correlations of relative action value and adaptability whereby z-scored values for RAV and adaptability index in the large disparity (15 vs. 0 μl) reward environment were correlated with values from the 3x (top) and 1.5x (bottom) relative reward environments. **(C)** We noted a significant correlation between the RAV for the large and 3x reward ratio and **(E)** a trend in the correlation of the large and 1.5x reward ratios. **(D,F)** We note no cross-contingency correlation for the adaptability index.

While manipulations of relative reward contingencies show consistent population effects on outcome valuation and behavioral flexibility, we sought to characterize whether these metrics exhibited stable patterns across multiple sessions and contingencies. For each session, we extracted the relative action value and adaptability index and z-scored these values relative to the population performing the same day of a given operant contingency. This allowed us to estimate the individual value sensitivity and behavioral flexibility of mice relative to the population for each session. We observed a significant correlation of relative action value and adaptability between the first day and subsequent sessions (averaged metrics from days 2 to 3) within the same reward contingency (Figures 6A,B). Next, we analyzed cross-contingency stability of these metrics, using performance in 15 vs. 0 μL as our baseline measures of reward sensitivity and behavioral adaptability. We noted a correlation in the relative action value with data produced from the 3x relative reward contingency (Figure 6C) and a trend in the 1.5x (Figure 6E), suggesting trait-like patterns of relative-reward sensitivity. We did not observe a significant non-zero correlation between behavioral flexibility at either relative reward ratio (Figures 6D,F). This suggests that while both metrics are stable across sessions within mice, the relative action value is also robust across relative reward ratios.

### Integration of Effort Costs and Benefit for Action Selection

**Figure 7:**
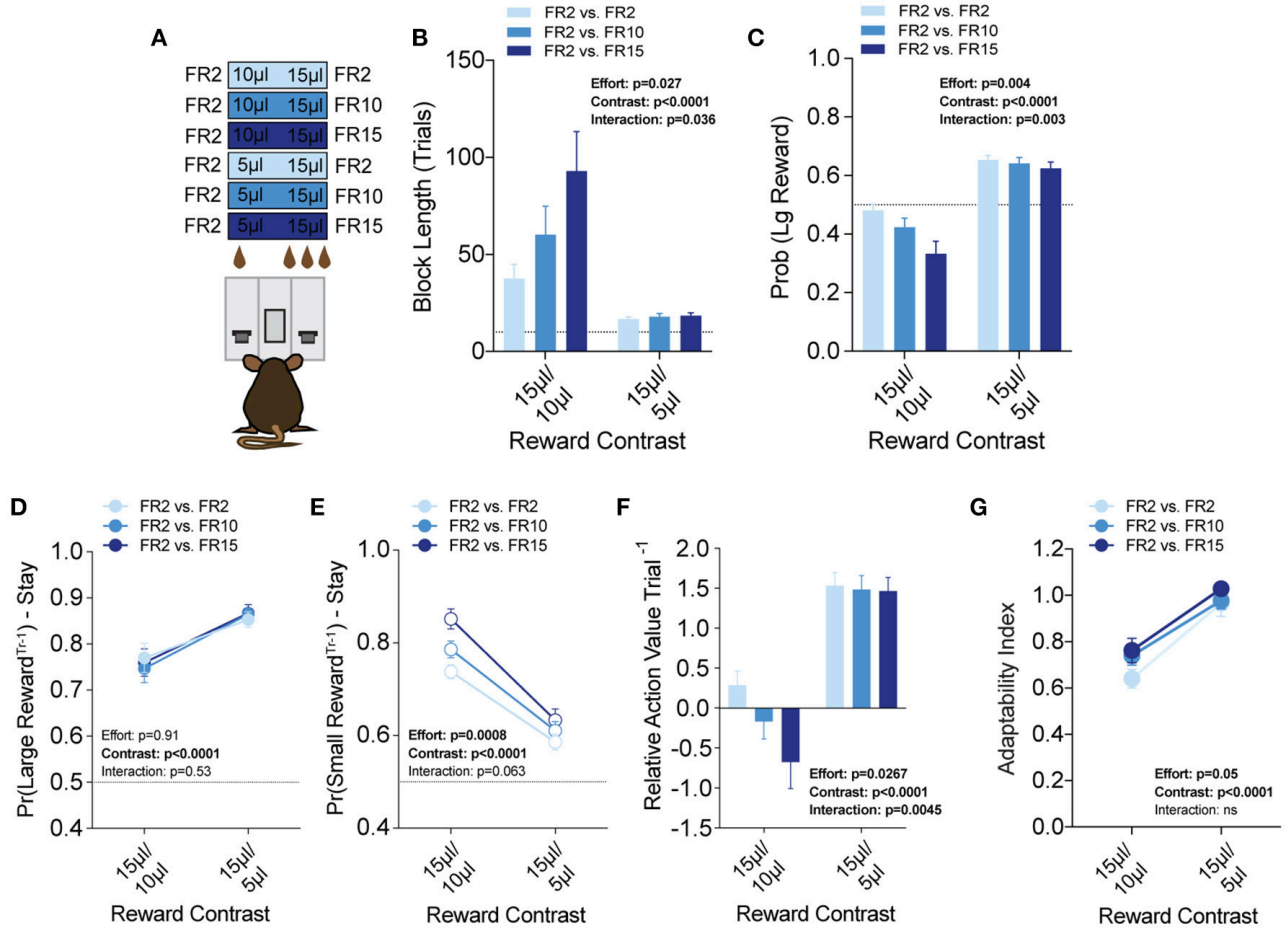
Effort costs alter the reinforcing properties of small reward alternatives**. (A)** Mice (*n* = 19) were tested at two reward magnitude contrasts across three different operant schedule contrasts. **(B,C)** Both the relative reward contrast as well as the effort schedule had a significant effect on the block length and the overall rate of selecting the large reward as well as a significant interaction between the effects of effort and the relative reward on both measures. **(D)** The imposition of high-effort costs on the large reward alternative did not have statistically significant effects on the reinforcing properties of that alternative. **(E)** Increased operant scheduling on the large reward alternative had a statistically significant effect on the reinforcing properties of the small reward choice. **(F)** We observed a significant interaction between reward contrast and effort, with increased effort costs exerting more dynamic effects in the small reward contrast environment. **(G)** Increased effort to reward had a small but significant effect on behavioral flexibility. Pairwise analysis indicates that behavioral flexibility was actually increased with the application of increased operant scheduling. All data analyzed by Repeated Measures (Reward Ratio, Effort) Two-Way ANOVA.

Efficient value-based choice requires integrating the positive benefits of an outcome with negative events that are associated with or required to obtain that outcome. To model outcome-associated costs in mice, we modified our operant contingencies to require increased physical effort to obtain the higher volume reward (Figure 7A). Within the context of our task, each lever was linked to a specific fixed ratio operant schedule to create three relative effort contrasts—fixed-ratio 2 (FR2) vs. FR2, FR2 vs. FR10 and FR2 vs. FR15. We tested each of these in 3x (15 vs. 5 μl) and 1.5x (15 vs. 10 μl) relative reward regimes (P_rew_ = 1), with the higher response schedule exclusively paired to the large reward alternative. Interestingly, global task measures (average block length, overall probability of large reward) demonstrated that disparities in effort schedule did not significantly alter the performance of mice in the task when the relative reward difference was sufficiently large (see 15/5 μl column in Figures 7B,C). In contrast, we observed a stepwise increase in the block length and decrease in large reward selection as the amount of required responses for the large reward increased in the regime with the smaller discrepancy in reward magnitude (see 15/10 μl column in Figures 7B,C). These changes in global performance were matched by changes in the relative action value of large and small rewarded outcomes (Figure 7F, left).

To better understand the effect of effort costs on the choice patterns observed, we analyzed and compared the reward-stay probabilities of animals after particular outcomes, as described above. Surprisingly, we found that while the increased response schedule was associated exclusively with the large reward alternative on any given trial, the distribution of mouse choice following a large reward was unchanged in either reward regime (Figure 7D). Instead, the increasing response requirement on the larger volume lever was associated with increases in the reinforcing property of the lower volume, but less effortful outcome in 15/10 μl but not 15/5 μl regimes (Figure 7E). Among mice that completed at least five blocks in each of the reward contingencies tested we observed a small but significant main effect of increasing effort on behavioral flexibility (Figure 7G).

### Integration of Temporal Delay Costs and Benefit for Action Selection

**Figure 8:**
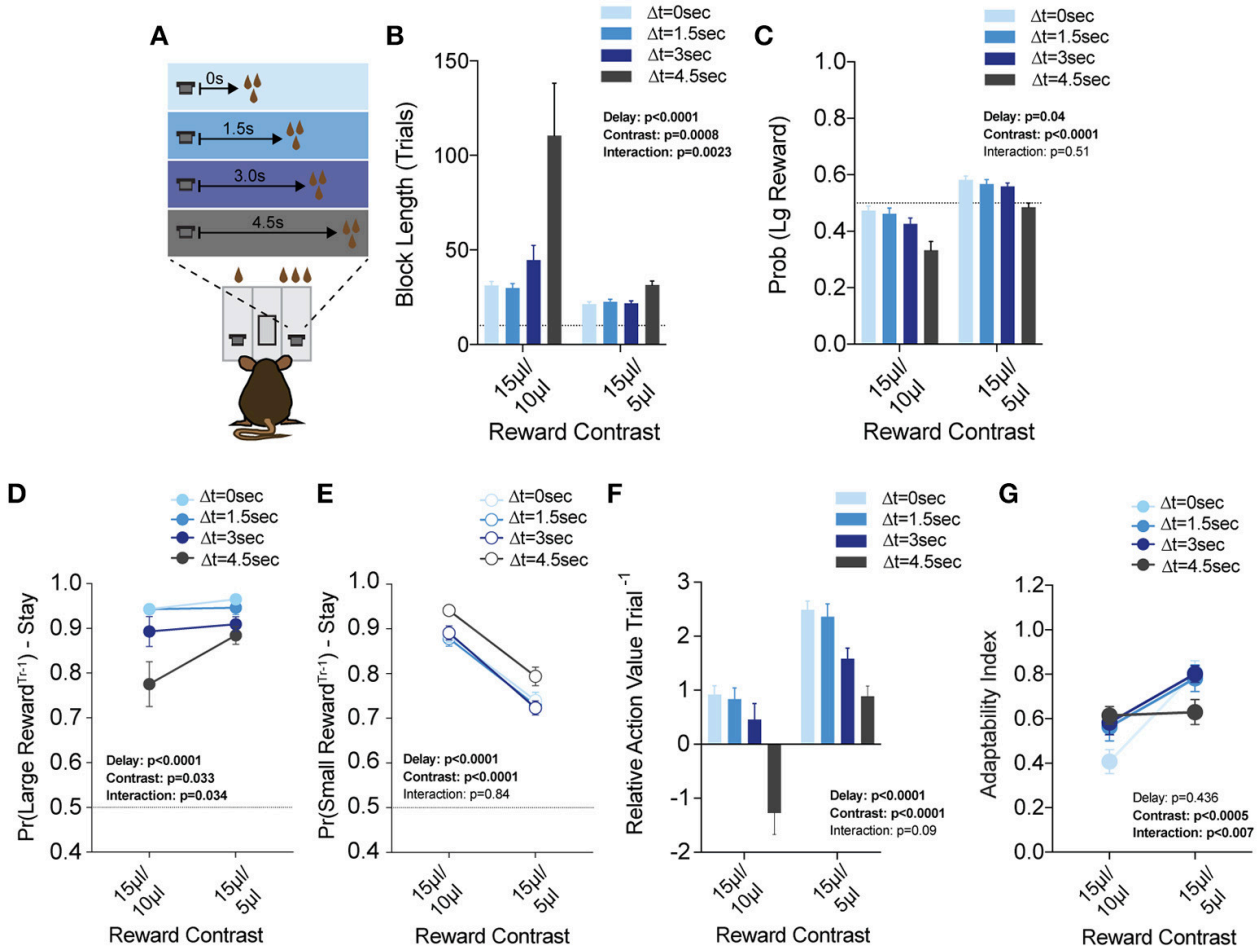
Delay costs primarily alter the reinforcing properties of large reward alternatives. **(A)** Mice (*n* = 21) were tested at two reward magnitude contrasts across four delays to reward delivery, applied exclusively to the large reward option. **(B,C)** Both the relative reward contrast as well as the delay to reward had a significant effect on the average block length and the probability mice chose the large reward over the course of a session. We observed a significant interaction between the effects of delay and reward contrast for block length, but not Pr(Large Reward). **(D)** The application of delay costs to the large reward alternative had a significant effect on win-stay behavior following large reward outcomes, where we observed an interaction between delay and reward contrast. **(E)** The addition of delay to large reward outcomes had a statistically significant effect on the reinforcing properties of small reward outcomes. Nevertheless, Tukey's multiple comparisons revealed no pairwise differences between values at three of the four delay regimes. **(F)** The relative reinforcing properties of large and small reward outcomes is sensitive to reward magnitude contrast as well as increasing temporal delay to reward delivery. **(G)** Increased temporal delay to reward had a significant effect on flexibility, with adaptability generally being higher with larger reward contrasts. All data analyzed by Repeated Measures (Reward Ratio, Delay) Two-Way ANOVA.

Given the unique manner in which effort costs interacted with relative benefits, we sought to test whether this observation held across other cost modalities that lower the value of a given choice. To do this, we introduced temporal disparities between choice and reward delivery (ΔT = 0, 1.5, 3, and 4.5 s) and applied these delays exclusively to large reward alternatives in any given block (Figure 8A). We again tested each of these delay environments in both large (15 vs. 5 μl) and small (15 vs. 10 μl) reward discrepancy environments (P_rew_ = 1). An analysis of global performance demonstrated main effects of relative reward ratio and temporal delay on Pr(Large Reward), and a significant interaction between these variables for block length (Figures 8B,C).

While the global effects of delay largely mirror the effects of effort (Figures 8F,G), we found this was achieved by distinct effects on choice patterns. Increasing the temporal delay of reward primarily altered the distribution of choices on the cost-associated alternative (the large reward) rather than the contralateral lower cost side, as in the effort paradigm (compare Figures 8D,E with Figures 7D,E). While we note a significant effect for delay in win-stay probability following both large and small rewards, further investigation of pairwise differences revealed that win-stay probabilities following small reward outcomes were unaltered at either reward regime for three of the four temporal delays tested (all but ΔT = 4.5 s), while multiple such pairwise differences exist in the win-stay behavior exhibited after high benefit-high delay outcomes.

### Interaction of Benefit and Cost Sensitivity in Goal-Directed Decision Making

**Figure 9:**
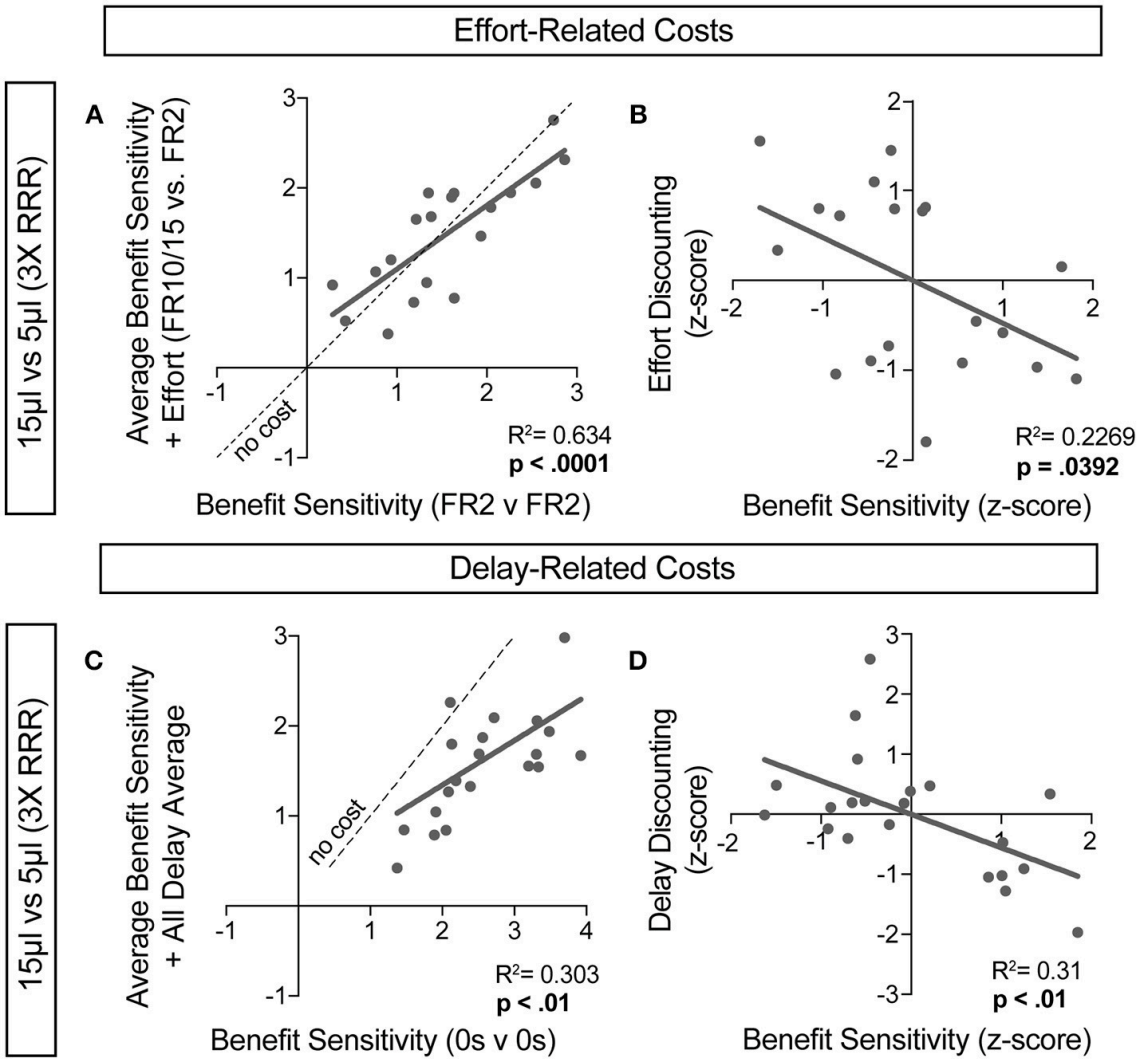
Sensitivity to reward benefits and costs are correlated. **(A,C)** The baseline sensitivity of mice to reward benefits (measured as the relative action value of 15 v 5 μL in FR2 v FR2 for effort **(A**, *n* = 19) and 0 s v 0 s for delay **(C**, *n* = 21) was correlated with the averaged relative action values measured upon addition of effort and delay costs. We found a significant correlation between the sensitivity of animals to reward benefits with and without the addition of associated costs, consistent with “trait-like” expression of reward sensitivity. **(B,D)** To quantify the extent to which each cost modality altered mouse choice distributions we took the difference in relative action value of mice in baseline conditions and with application of operant costs (RAV_cost_ -RAV_baseline_). Increasing negative values indicate larger choice disruption in the presence of costs. We observed a significant relationship in the sensitivity of mice to reward benefits and the sensitivity of mice to the addition of reward costs, relative to the population.

**Figure 10:**
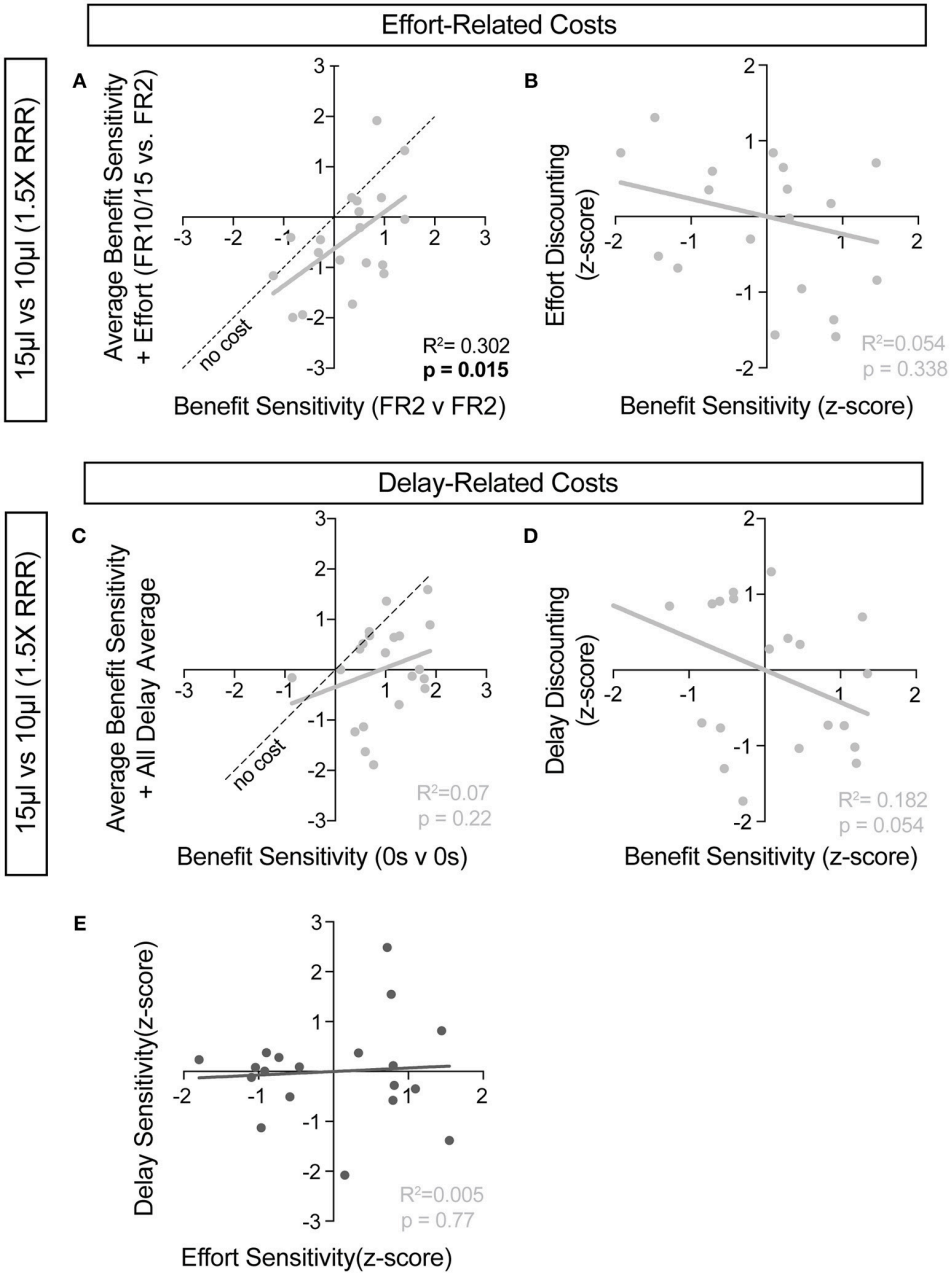
Cost-benefit correlations with small reward contrats. **(A,C)** The sensitivity of mice to reward benefits was measured as the relative action value in baseline conditions for the effort **(A**, FR2 v FR2, *n* = 19) and delay **(C**, 0 s v 0 s, *n* = 21) experiments (Reward Contrast: 15 v 10 μL). These values were correlated with the averaged relative action values measured in mice with the addition of effort and delay costs. **(A)** At the low discrepancy in reward magnitude, there is a significant correlation between the sensitivity of animals to reward benefits, in environments with and without the addition of increased operant scheduling. **(C)** We note no cross-session correlation in reward sensitivity with the application of temporal delay costs in a reward environment with a small discrepancy in reward benefit. **(B,D)** We note a significant relationship in the sensitivity of mice to reward benefits and the sensitivity of mice to the addition temporal delay **(D)** but not effort costs, relative to the population, in this reward environment. **(E)** No correlation exists between the sensitivity of mice to the two cost modalities tested.

For further insight into any systematic relationships between how animals process benefit and cost, we analyzed individual mouse values for benefit sensitivity, with and without relative cost considerations. Our previous data demonstrated trait-like patterns of relative reward bias, reflecting an underlying distribution of reward sensitivities (Figure 6). We hypothesized that these patterns would persist in the face of costs, with the animals most sensitive to relative reward benefit showing the least alteration in choice pattern upon introduction of barriers to reward. To test this for both cost modalities, we took the relative action value at baseline conditions (Effort: FR2 v FR2; Delay: 0 s v 0 s) and measured how this value was altered in the presence of reward-associated costs. Significant correlations between the sensitivity to reward of mice with and without relative costs confirmed that the addition of cost did not dramatically shift the rank order of benefit sensitivity within the whole population for large reward contrasts (i.e., the most reward sensitive animals continued to be so in the presence of increasing cost) (Figures 9A,C). Similar trends were observed when comparing the noisier small reward contrast data (Figures 10A,C). Nevertheless, an analysis of the relationship between benefit sensitivity and cost sensitivity (defined as the magnitude of negative modulation of RAV by cost, see Methods) demonstrated that both effort and delay cost most dramatically altered the choice pattern of mice that were most sensitive to relative reward benefits in baseline conditions (Figures 9B,D, 10B,D). For example, mice that were most sensitive to larger reward volume (large positive z-scores on benefit) in general exhibited the largest reduction in RAV once costs were introduced (most negative z-scores on effort discounting), yielding data points in the lower right quadrant (Figure 9B). In addition, we noted no correlation in the sensitivity of mice to the different cost modalities (Figure 10E).

## Discussion

The development of quantitative behavioral assays in mice that probe core features of value-based action selection is an important step toward understanding the neural substrates underlying economic decision-making. These circuits are of critical behavioral relevance, regulating how animals select actions based on the assigned values of available options, a fundamental organizing principle for how organisms interact with their environment (Knutson et al., 2005; Lau and Glimcher, 2008; Hunt et al., 2012; Allen et al., 2017). As such, exploring their function may eventually illuminate the pathophysiological underpinnings of goal-directed dysfunction in neuropsychiatric disorders, a symptom domain that severely limits societal function. As mice currently allow the highest level of experimental interrogation within mammalian systems (Jaramillo and Zador, 2014), it is essential to have a thorough understanding of how they make economic decisions, as well as the extent of individual animal variability in these processes. This paper details a set of behavioral paradigms in mice that captures essential elements of economic choice behavior, including the sensitivity to differences in total value, flexibility to action-outcome contingency shifts, and outcome-sensitive modulation of task engagement.

### A Paradigm to Assess Value-Based Choice in Mice

Our goal was to create a paradigm that could produce robust quantitative assessments of value-based choice. We reasoned such measures would result from observing a large number of choices over a range of benefit and cost environments. To achieve this, we employed a repeated trial structure in which groups of trials with the same contingency were arranged into blocks, and contingencies were regularly shifted in response to proximal patterns of mouse choice behavior. By dynamically alternating reward contingencies upon the detection of prolonged choice bias, we ensured that mice remained continually engaged in updating subjective values for choice alternatives. To further maintain reward-sensitive behavior, we reduced reward probability to increase exploratory choice. Our own data confirms the necessity of these approaches, as fixed contingency protocols and certainty of reward delivery led to highly biased responding, even in the absence of value differences between the two levers (Figure 2A). In order to isolate the ability of mice to create and act exclusively upon outcome valuations, our task de-emphasized the use of audio or visual cues in signaling action-outcome relationships or contingency alteration. Instead, we believe, the paradigm forces mice to use reward feedback to shape internal representations of option value, which are then externally expressed as a distribution in choice behavior. In such an environment, we argue, the most accurate behavioral readout of the internal representation of outcome value is the probability that mice return to or “stay” on an alternative after having just received that outcome.

### Quantification of Value-Based Choice

In highly trained mice, our logistic regression models demonstrate heavy discounting of all but the trial immediately preceding the current choice (Figure 1F). Effectively, this task shaped mice to adopt an enduring “win-stay, lose-shift” strategy that heavily favored the most proximal reward outcomes. We attribute this narrow integration window to the specifics of our paradigm as opposed to fundamental constraints of mouse working memory, as alternative behavioral frameworks in mice demonstrate integration of up to three trials in the past (Tai et al., 2012; Parker et al., 2016). Nevertheless, this observation provided a framework for understanding local decision-making strategies in this task and provided a framework for subsequent analysis. Having established an understanding of the cognitive strategies mice used to seek reward in this paradigm, we developed metrics for outcome sensitivity (relative action value), behavioral flexibility in new environments (adaptability index) and modulation of action performance (relative initiation latency). The relative action value is a measure of the relative reinforcing properties of two competing operant outcomes. By comparing choice distributions following discrete outcomes at the individual-mouse level, this measure minimized individual mouse variability in non-outcome-related stay behavior. The adaptability index takes advantage of the behavioral constraint dictating that each behavioral “block” ends in a similar pattern of choice. By constraining the pre-switch behavior in this way, we create a fixed behavioral state to contextualize choices made after contingency switches. We noted that a significant amount of the variability (>80%) in mouse performance (measured by the rate of large reward selection) in any individual session could be explained by a combination of the animal's sensitivity to reward discrepancies and their behavioral flexibility.

To complement our regression-based behavioral analysis, we submitted our data to standard Q-learning models (Figure 5) (Katahira, 2015). This approach can statistically disambiguate how effectively recent outcomes modify action-values (α) as well as how much these value differences drive choice patterns (β). We found that α was consistently higher than 0.79 across all relative reward contrasts and probabilities, supporting our conclusion from regression models that well-trained animals rely almost exclusively on one prior trial for feedback-guided choice. Similarly, we found similar β parameters across test conditions, suggesting that mice did not substantially alter their exploration-exploitation strategies. We did note that choice persistence (returning to the same choice regardless of outcome) changed with contingency, being higher in reward-rich environments (high P_rew_, large rewards on both levers). Overall, these data suggest that the changing choice patterns observed during relative reward experiments are not likely due to fundamental changes in how mice approach the task but instead to the nuanced dynamics of the action values themselves. If true, this behavioral paradigm should provide an ideal testing ground to examine the cellular integration of outcome cost and benefit on action values during decision making.

### Trait-Like Expression of Behavioral Characteristics

To further establish the validity of these measures and their stability within animal, we examined their cross-session and cross-contingency consistency. We reasoned that value processing likely represents a behavioral trait—a temporally stable behavioral pattern unique to each subject. In accordance with this, we observe significant within-animal consistency across 3 days of repeated contingencies, for both relative action value and adaptability (Figures 6A,B). As a further test of trait-like stability, we assessed whether similar reward sensitivity and flexibility metrics would manifest across multiple relative reward regimes. Here we noted that relative action value at 15 vs. 0 μl was significantly correlated to these measures obtained at 15 vs. 5 μl and trended toward a correlation at 15 vs. 10 μl. In contrast, flexibility at 15 vs. 0 μl did not correlate with these values at other reward ratios. Consistent with this, several studies have shown high variability in the cognitive flexibility of mice due to moderate stressors (Graybeal et al., 2011), which can be differentially induced via handling (Sorge et al., 2014), degree of food deprivation (Heiderstadt et al., 2000), and housing conditions (Tanimura et al., 2008). In sum, individual-animal analyses indicate that with sufficient training, mice can reproducibly perform complex value-based tasks typically reserved for other model systems (Gold and Shadlen, 2007; Jaramillo and Zador, 2014). The cross-session reproducibility of these reward metrics suggests that we are extracting meaningful derivations of elements of mouse reward processing, and that single sessions with >200 trials may be sufficient to generate individual representative values in any given reward contingency. This reliability, together with the discrete temporal structure of our task, will make it especially suited for combination with *in vivo* physiological recordings of corresponding neural activity.

### Economic Decision-Making in our Task

Within this quantitative framework of mouse behavior, we sought to answer a fundamental neuroeconomic question: How do the benefits and costs of rewarded outcomes shape mouse behavior? Characterizing how diverse features of mouse behavior are modulated by value is a critical step in elucidating neural circuits with specific reward processing function. We observed that mice differentially altered their choice patterns in response to the relative magnitude of the previously rewarded outcome—with more extreme distributions for outcomes of higher benefit (Figure 3F). Further analysis revealed that the magnitude of proximal rewards not only altered mouse choice distributions, but also had a significant effect on focal task engagement, suggesting short-term circuit modifications in response to reward have effects not just on choice patterns, but also action execution (Figure 3H). We additionally noted that that the ability of mice to flexibly adapt their behavior scales with the relative magnitude of rewarded outcomes (Figure 3I).

We then associated two cost modalities exclusively with high-benefit options in environments with large and small disparities in reward, to assess the integration of reward costs in decision making. We demonstrate that in environments with large differences in reward, costs associated with selecting those outcomes are heavily discounted. Surprisingly, while increased operant scheduling and temporal delay both decrease the relative value of choice alternatives, we found that these two cost modalities differentially altered the relative value of previous outcomes, with effort increasing stay behavior on the small reward and delay reducing stay behavior on the large reward at the population scale (compare Figures 7D,E with Figures 8D,E). This finding suggests potentially unique circuit mechanisms underlying the subjective valuation of these choice costs and provides further evidence of the sophisticated value judgments mice can perform. Deeper analysis of our data demonstrated interesting interactions between benefit and cost sensitivity at the individual animal level. We found that the mice whose choice distributions were most radically altered by the addition of costs were the same animals exhibiting the highest sensitivity to differences in rewarded outcome (Figure 9). This observation raises the interesting possibility of common circuit mechanisms for controlling processing of both components of value computation.

### Conclusions and Considerations

In summary, these findings demonstrate the sensitivity of our behavioral assay to decision-making strategies adopted by mice during economic choice, while revealing stable, mouse-intrinsic differences in value-based action selection. An important direction for future work will be to characterize the local circuits governing the distinct behavioral features described here. For example, the orbitofrontal cortex is intimately involved in value representations central to efficient performance of this task (Schoenbaum et al., 2011; Gourley et al., 2016; Baltz et al., 2018). As updated reward value is integrated into multiple elements of the decision-making process—including choice bias, adaptability, and task engagement—it will be important to ask whether orbital cortex mediates these functions via distinct subcortical circuits for motor control. Furthermore, these paradigms will prove essential in determining how cost-benefit calculations are encoded at the cellular level within striatal circuits (Mikhael and Bogacz, 2016).

We believe that the reliability and robust quantitative nature of these paradigms makes them well-suited to investigating the complex issue of how reward processing is altered by environmental and genetic factors. We uncovered a substantial amount of between-animal variability for value processing, perhaps surprising given the genetic homogeneity of the mouse strain used. We believe this observation is both unsurprising and fascinating. In our view, genetically encoded information provides a basic blueprint for the assembly and maintenance of neural circuits. However, quasi-stochastic processes such as axonal targeting and sub-cellular synapse localization are then superimposed on this basic plan, generating diversity within circuits, and behavior. These differences can be further amplified by existing social hierarchies and other experiences in the home cage leading up to testing (Greenberg et al., 2014; Porcelli and Delgado, 2017). Given this complexity, our ability to ascribe circuit-specific genetic contributions to reward processing abnormalities necessitates the type of stable, robust metrics generated by this work. While we acknowledge the possibility that such stable “trait”-like reward-sensitivity characteristics may in fact reflect task-specific behavioral patterns, their reproducibility provides solid foundation for further systems-level analyses (Farashahi et al., 2018). As such, these paradigms may provide novel pathways for analyzing reward processing in mouse genetic models for neuropsychiatric disease.

## Author Contributions

OA performed operant experiments, analyzed data, generated analysis code, and wrote the manuscript. MPF performed operant experiments. MVF conceived the project, analyzed data, and wrote the manuscript.

### Conflict of Interest Statement

The authors declare that the research was conducted in the absence of any commercial or financial relationships that could be construed as a potential conflict of interest.
